# Polymer-stabilized palladium nanoparticles for catalytic membranes: *ad hoc *polymer fabrication

**DOI:** 10.1186/1556-276X-6-406

**Published:** 2011-06-01

**Authors:** Berta Domènech, Maria Muñoz, Dmitri N Muraviev, Jorge Macanás

**Affiliations:** 1 Analytical Chemistry Division, Chemical Department, Universitat Autònoma de Barcelona, 08193 Bellaterra, Barcelona, Spain; 2 Department of Chemical Engineering, UPC, C/Colom, 1, 08222 Terrassa, Barcelona, Spain

## Abstract

Metal nanoparticles are known as highly effective catalysts although their immobilization on solid supports is frequently required to prevent aggregation and to facilitate the catalyst application, recovery, and reuse. This paper reports the intermatrix synthesis of Pd^0 ^nanoparticles in sulfonated polyethersulfone with Cardo group membranes and their use as nanocomposite catalytic membrane reactors. The synthesized polymer and the corresponding nanocomposite were characterized by spectroscopic and microscopic techniques. The catalytic efficiency of catalytic membranes was evaluated by following the reduction of *p*-nitrophenol in the presence of NaBH_4_.

## Introduction

The unusual physical, chemical, and catalytic properties of metal colloids (better known in nowadays as metal nanoparticles, MNPs) have attracted increasing interest of scientists and technologists during the last decade [1]. A characteristic high percentage of surface atoms and the associated quantum effects make MNPs efficient and selective catalysts for several types of catalytic reactions [2,3]. However, for most practical catalytic applications, MNPs must be immobilized on solid supports to prevent their aggregation and to facilitate the catalyst recovery [4]. In this sense, encapsulation of MNPs in polymers seems advantageous because, in addition to stabilizing and protecting effects towards MNPs, polymers offer unique possibilities for enhancing the access of reactants to the catalytic sites [5]. The *in situ *synthesis of MNPs into a polymeric membrane matrix permits to combine catalysis and membrane processes, which may result in process intensification [6,7]: destruction and separation of pollutants in a single step [7,8]. These polymer stabilized MNPs (PSMNPs) can be synthesized by various methods [1,9,10], including the intermatrix synthesis (IMS) technique [11,12]. Some common polymers such as cellulose acetate, hydrophilized polysulfone, polyacrylic acid-modified polyethersulfone, polyvinylidene fluoride, and many others, which are widely used as membrane materials, have been successfully employed for the synthesis of PSMNPs [13]. These polymers can be used as a support of metal catalysts [14,15] after a simple casting procedure. However, it is imperative to adequate the polymeric matrix for both the IMS of PSMNPs and the final application of the nanocomposites. Therefore, the development of catalytic membranes requires the use of polymers with good chemical, thermal, and mechanical stability, and these polymers also have to be easily fabricated in a suitable form for their further practical application [16].

**Figure 1:**
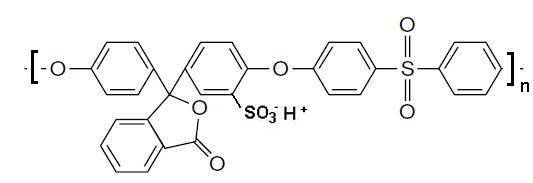
**Structural unit of sulfonated polyethersulfone with Cardo group (SPES-C) in its acid form**.

The application of the IMS technique implies that the matrix polymer must bear some functional groups (e.g., sulfonic or carboxylic) capable to bind metal ions (PSMNP precursors). Yet, the presence of these groups is known to increase the hydrophilicity of the polymer, which can hinder, in some cases, the preparation of membranes with the required properties. For example, sulfonated poly(etheretherketone) has already proved to be a very suitable matrix for IMS of various PSMNPs [11,12], but it cannot be used for the preparation of catalytic membranes by phase inversion technique^15 ^due to the following reasons: (1) its high hydrophilicity and (2) quite low mechanical stability. For this reason, a compromise needs to be found for each type of application by balancing hydrophilicity and hydrophobicity. To cope with this drawback, we propose the sulfonation of polyethersulfone with Cardo group (PES-C) which has a very hydrophobic group in its skeleton (a five-member lactone ring of a phenolphthalein moiety) but whose sulfonation can be done in a simple way [16]. The resulting structure of sulfonated PES-C (SPES-C) is shown in Figure 1.

The main goal of this work is the development of palladium nanoparticles (Pd^0^-NPs) by IMS technique inside SPES-C membranes for their use as catalytic membrane reactors. The characterization of the catalytic effect was performed by following the reduction of *p*-nitrophenol (4-np) in presence of NaBH_4 _[6].

## Experimental

### Materials

Commercial PES-C was kindly supplied by Dr. Trong Nguyen from Université de Rouen, France. H_2_SO_4_, HCl, *N*-methyl-2-pyrrolidone (NMP), and *N*,*N*-dimethyl-formamide (DMF) (all from Panreac, S.A., Castellar del Vallès, Spain) organics and metal salts (4-np, NaCl, NaOH, NaBH_4_, CuSO_4_, Na_2_SO_4_, and [Pd(NH_3_)_4_]Cl_2 _all from Aldrich, Munich, Germany) were used as received.

### PES-C sulfonation

Sulfonation of PES-C was carried out following a procedure similar to those published elsewhere [16,17]. The polymer powder was dried at 70°C for 48 h prior to use. Then, it was dissolved (11% *w*/*v*) in concentrated sulfuric acid and mixed at constant temperature (60°C). After 5 h, the reaction medium was precipitated in a cold-water bath under strong stirring to obtain the SPES-C in its acid form. Afterward, it was neutralized with NaOH 1 M solution, filtered, washed with deionized water, and dried at 70°C (polymer in Na form).

### Membrane casting

Membranes were prepared by wet phase inversion method [15] by using a polymer solution in NMP (25 wt.%). After casting, membranes were stored in deionized water.

### Synthesis of Pd^0^-NPs

The synthesis of Pd^0^-NPs in SPES-C membranes was carried out by a two-step procedure (see below Equations 1 and 2) [11,12], consisting in the loading of sulfonic groups with [Pd(NH_3_)_4_]^2+ ^ions and their subsequent chemical reduction inside polymeric matrix by using NaBH_4 _0.1 M solution:(1)(2)

### Polymer and nanocomposite characterization

Attenuated total reflectance Fourier transform infrared (ATR-FTIR) spectra were recorded with a Perkin Elmer Spectrum GX spectrometer (Norwalk, CT, USA).

Membrane water uptake (MWU, g H_2_O/g dry membrane) was determined by a simple procedure: membrane samples stored in water were weighed (*W*_w_), then dried in the oven for 48 h at 80°C, and weighed again (*W*_d_). MWU was calculated by using Equation 3:(3)

The ion-exchange capacity (IEC) defined as the number of milliequivalents (meq) of ionogenic groups (e.g., SO_3_^-^) per gram of dry membrane (*W*_d_) was determined by two different methodologies:

1. Indirect titration: samples were equilibrated with HCl 2 M for 24 h to convert the functional groups of the polymer into protonated form. Then, samples were equilibrated with an excess of NaCl 1 M solution for 24 h, and the resulting solutions were titrated with NaOH 4.3 mM using phenolphthalein as indicator.

2. Cu^2+ ^displacement and determination: a sample of membrane in the H form (see above) was equilibrated with 20 ml of CuSO_4 _0.1 M solution for 20 h, washed with water several times and finally equilibrated with 20 ml of Na_2_SO_4 _0.5 M. The exchanged Cu^2+ ^ions were analyzed by inductively coupled plasma-atomic emission spectroscopy (ICP-AES) using an Iris Intrepid II XSP spectrometer (Thermo Electron Corporation, Milford, MA, USA).

The commonly used sulfonation degree^18 ^(SD), which corresponds to the molar ratio of sulfonated units to the total basic units, was calculated from IEC value by Equation 4 where *M*_0 _was 532 g/mol, and the molar mass variation (considering both the sulfonation and the conversion from H to Na form) was 102 g/mol:(4)

In order to determine the exact metal content in the prepared nanocomposites, the membrane samples of known weight were treated with *aqua regia *to completely dissolve all MNPs. The resulting solutions were diluted and analyzed by ICP-AES. The average uncertainty was <2% in all cases.

### Characterization of Pd^0^-NPs

With the aim of characterizing the size and structure of Pd^0^-NPs, transmission electron microscope (TEM) images were obtained by using a JEOL JEM-2011 Microscope (Jeol Ltd., Tokyo, Japan). For this purpose, a drop of samples diluted in DMF was deposited onto a Cu TEM grid, followed by solvent evaporation at room temperature.

### Catalytic properties evaluation

The catalytic performance of modified SPES-C membranes was evaluated by using the reduction of *p*-nitrophenol (4-np) by NaBH_4 _to *p*-aminophenol as a model reaction [7], which was considered to follow the pseudo-first-order kinetics. In a typical run, a given amount of NaBH_4 _was added to 5 ml of 4-np solution (5 mM) to achieve a NaBH_4 _concentration of 55 mM. After mixing, a piece of nanocomposite (1 cm^2^) was added to the vessel. The process was monitored at 390 nm by a Pharmacia LKB Novaspec II spectrometer (Biochrom Ltd., Cambridge, UK).

## Results and discussion

**Figure 2:**
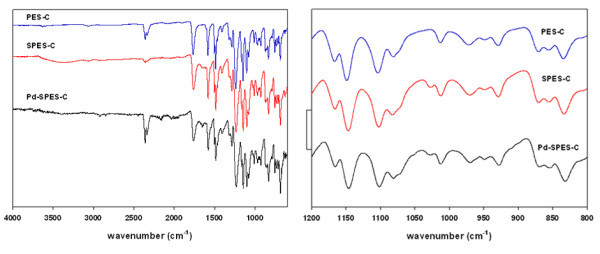
**ATR-FTIR spectra of PES-C, SPES-C, and Pd**^**0**^**-NP-SPES-C (left)**. Partial spectra corresponding to 800 to 1,200 cm^-1 ^interval (right).

The ATR-FTIR-ATR spectra of both bare (PES-C) and sulfonated polymer (SPES-C) shown in Figure 2 have been used to follow the effectiveness of the sulfonation procedure. As it is clearly seen, spectra only differ by the peak at about 1,030 cm^-1 ^assigned to symmetric S = O stretching due to the introduction of -SO_3_H groups into the polymer chains. This fact confirms that the rest of the polymer structure is not degraded in the course of sulfonation. As it is also seen in Figure 2, the ATR-FTIR spectrum of the Pd^0^-NMP containing nanocomposite also shows the same peak.

The qualitative confirmation of polymer sulfonation was also supported by the quantitative determination of the IEC and the corresponding SD values. The IEC value obtained by titration of three samples (uncertainty 95%, *t *= 2.92) was 0.48 ± 0.12 meq/g whereas that obtained by Cu^2+ ^exchange was found to be equal to 0.31 ± 0.07 meq/g. The higher value obtained by the first method can be due to incomplete displacement of Cu^2+ ^from the polymer and to the partial retention of some metal ions in the matrix when preparing samples for ICP-AES. The obtained SD values (0.27 ± 0.09 and 0.17 ± 0.05, respectively) are far from the practical limitation of 0.9, which has been reported to cause the polymer dissolution in water [18]. These results prove that a moderate SD can be achieved under mild reaction conditions corresponding to reasonable reaction temperature and time (60°C, 5 h) just by using concentrated H_2_SO_4_. This procedure appears to be far simpler than, for instance, the polymer modification by the metalation route [19].

The measured values of MWU for the sulfonated polymer and the corresponding nanocomposite were found to be 2.73 ± 0.13 and 3.21 ± 0.19 g H_2_O/g dry membrane, respectively (three replicates in each case). These values prove that (1) even if hydrophilicity of the material is high, it does not result in the membrane dissolution, and (2) the IMS of Pd^0^-NPs does not substantially affect the membrane morphology and the accessibility of water to the sulfonic groups.

The content of Pd-MNPs in the nanocomposites was found to be 156.7 ± 0.8 μmol of Pd^0 ^per gram of dry nanocomposite (16.68 ± 0.08 mg Pd^0^/g) by analyzing eight different samples by ICP-AES. Similarly to the other studied parameters, it is worth noting the high homogeneity of samples, probably due the IMS methodology.

**Figure 3:**
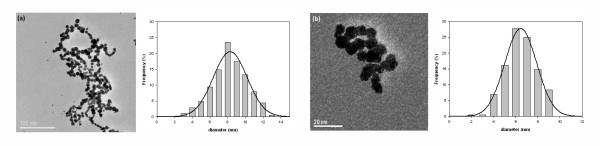
**TEM images and size distribution histograms of two Pd**^**0**^**-MNP samples prepared in the same way**.

Figure 3 shows typical TEM images of Pd^0^-NPs synthesized inside SPES-C matrix and their size distribution histograms. As it is seen, Pd forms almost spherical nanoparticles characterized by quite narrow size distribution with the most frequent diameters lying inside the 4- to 8-nm range. Particles are well separated from each other, which testifies to the high stabilizing efficiency of SPES-C matrix towards Pg-MNPs.

**Figure 4:**
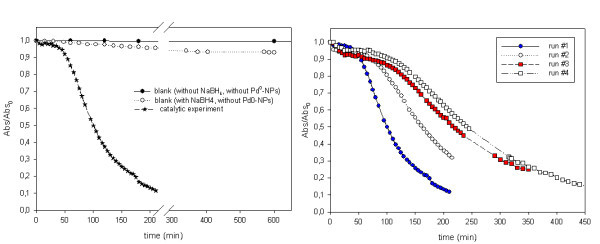
**Catalytic performance of Pd-MNPs containing membranes (*M***_**0 **_**sample)**.

The results of the catalytic characterization of samples are shown in Figure 4. As it is seen, MNP-free membranes do not exhibit any catalytic activity while the membrane samples containing Pd^0^-NPs show a clearly pronounced catalytic effect, which is confirmed by a quite fast absorbance decay. However, the attenuation only followed a linear trend after approximately 1 h. In order to understand this issue, it is important to consider that Pd is a classical hydrogen-storage metal [20,21]. Thus, the observed induction period can be associated with hydrogen loading (evolved from the decomposition of NaBH_4_) into the Pd-NPs, which competes with the catalytic reaction. Once the absorption of hydrogen has reached a saturation value (after the induction/activation period), the catalytic reaction prevails and the reaction rate follows pseudo-first-order kinetics at high extend. In fact, Pd^0^-NPs can already be partially loaded by hydrogen after the second IMS step (see Equation 2).

From a practical point of view, more than 90% of [4-np] can be easily reduced within less than 4 h through a single reaction step. This can be considered as the experimental confirmation of the suitability of this kind of catalytic membranes for decontamination applications. An apparent rate constant (*k*_app_) of [4-np] reduction has been estimated by assuming pseudo-first-order kinetics (see Equation 5). The *k*_app _value was found to be 0.0132 ± 0.0002 s^-1^.(5)

The possibility to reuse nanocomposite catalytic membranes was also evaluated. Figure 4 (right) shows the decrease of the normalized absorbance vs. time for four consecutive cycles, which testifies to the decrease of the reaction rate and the increase of the activation time after each cycle. This can be explained either by possible poisoning of the catalyst due to the generation of by-products of the 4-np reduction, which can interfere with the catalyst surface [7], or (and most probably) by a stronger competition between hydrogen absorption and catalytic reactions, after the initial discharge in the first cycle. It must be pointed out that the loss of catalytic activity cannot be explained by the leaching of Pd^0^-NPs from the matrix to the aqueous solution as no change of Pd content in nanocomposite membranes before and after catalytic cycles has been detected. Moreover, the absorbance of reacting solutions did not keep decreasing after the catalytic membrane was removed, even when stored overnight.

It has been reported that some polymer-encapsulated catalytic MNPs do aggregate after catalytic reactions [22]. However, due to the presence of the polymeric matrix, this mechanism might not be applicable for Pd^0^-SPES-C nanocomposites.

As it can be seen in Figure 4 (right), the decrease in catalytic activity diminishes after each catalytic cycle if one takes into account the slope of the absorbance vs. time plot. The difference between the first and second cycles is much higher than that corresponding to third and fourth cycles. This fact may suggest that after few cycles, the catalytic efficiency could be preserved, although at a lower value when compared with the first run. However, this point requires a stricter experimental confirmation if membranes operate under a pressure gradient.

## Conclusions

Polyethersulfone with Cardo group was easily sulfonated and used as a suitable matrix for IMS of palladium nanoparticles. Pd^0^-NPs were well separated from each other, which indicates the high stabilizing efficiency of the polymer. Synthesis of Pd-MNPs by IMS resulted not only in the formation of catalyst NPs but also in their additional activation due to dissolution of H_2 _inside Pd-MNPs. The membrane morphology is not affected by IMS of Pd-MNPs inside the polymeric matrix; however, further investigations are required to determine if the membrane transport processes are affected by the presence of MNPs. The obtained nanocomposite material exhibits a high catalytic activity, although an induction period is needed before reaction starts and the catalytic efficiency is not maintained with time. Further experiments are required in order to explain the deactivation effect observed when running consecutive catalytic cycles.

## Competing interests

The authors declare that they have no competing interests.

## Authors' contributions

BD carried out the experimental part concerning the polymer preparation and characterization, the nanocomposite synthesis and characterization, and the catalytic evaluation. BD also contributed in drafting the manuscript.. MM and DNM participated in the interpretation of the results. JM conceived the study, participated in its design and coordination, and wrote the main part of the manuscript. All authors read and approved the final manuscript.
